# A Multimodal Non-invasive Approach for Intracranial Pressure Assessment: A Single-Center Study

**DOI:** 10.7759/cureus.98744

**Published:** 2025-12-08

**Authors:** Dana Klavansky, Helaina Lehrer, Aris Desai, Gabriela Keeton, Neha Dangayach, Alexandra Reynolds, Spyridoula Tsetsou

**Affiliations:** 1 Neurocritical Care, Mount Sinai Hospital, New York, USA; 2 Neurocritical Care, Northwell Health, Manhassett, USA; 3 Neurocritical Care, Baylor College of Medicine, Houston, USA; 4 Neurocritical Care, Hackensack University Medical Center, Hackensack, USA

**Keywords:** acute brain injury, multi-modal diagnostics, neurology and critical care, non-invasive monitoring, secondary intracranial hypertension

## Abstract

Background: Intracranial pressure (ICP) monitoring is an integral part of acute brain injury management. While invasive ICP monitoring is the gold standard, there are several medical conditions that preclude its placement. The aim of the present study is to validate a multimodal approach for increasing ICP detection.

Material and methods: In this retrospective study, patients with acute brain injury who had an external ventricular drain (EVD) placement were included. We measured bilateral optic nerve sheath diameter (ONSD) and assessed for optic nerve disk elevation (ONDE) by using ocular ultrasound, bilateral middle cerebral artery pulsatility index (PI) by using transcranial Doppler, and assessed pupillary reactivity with or without a pupillometer as part of a multimodal assessment. We assessed the correlation and agreement of these values with the ICP measured by the EVD.

Results: A total of 56 measurements from 40 patients were included. The presence of three or more variables among mean ONSD > 5 mm, unilateral or bilateral presence of ONDE, at least one MCA PI > 1.2, and bilateral unreactive pupils demonstrated excellent specificity (1, 95%CI 0.92 - 1) and positive predictive value (PPV) (1, 95%CI 0.39-1) for association with ICP ≥ 15 mm Hg, with area under receiver operating characteristic curve of 0.84 (95%CI 0.74-0.94).

Conclusion: Non-invasive multimodal assessment can be easily done at the bedside and seems to correlate well with higher ICP.

## Introduction

Elevated intracranial pressure (ICP) is a common and severe complication after acute brain injury [1]. ICP-driven acute brain injury management with invasive ICP monitoring remains the gold standard according to the Brain Trauma Foundation [2] and Neurocritical Care Society [3,4], particularly when the patient’s Glasgow Coma Scale Score (GCS) is less than 8, the patient has an abnormal CT head, or if they meet two or more of the following criteria: greater than 40 years old, unilateral or bilateral motor posturing, and systolic blood pressure less than 90 mmHg. Invasive ICP monitoring can be placed via a burr hole into the ventricles, in the brain parenchyma, or epidurally/subdurally, with the external ventricular drain (EVD) being preferred as it offers both global ICP measurement and treatment for intracranial hypertension by cerebrospinal fluid diversion [5].

Even though invasive ICP monitors can be safely placed at the bedside by trained staff, devastating complications may occur [6]. However, irrespective of the risks, some institutions may still place these invasive monitors. Furthermore, their placement is precluded in many pathological conditions, such as in acute liver failure or other medical conditions with severe coagulopathy [7-9]. Under these conditions, acute brain injury management is not optimal and is often based on neurological examinations performed under sedation. Other complications, such as mispositioning, infection, and diminished accuracy for asymmetric hemispheric lesions, may also occur [10]. Furthermore, placement of these devices may take time and consume resources in more community-type settings [11]. Brain imaging in this population can be complex due to hemodynamic instability precluding transport, concern for elevated ICP limiting the duration the patient can lie flat, and the need for staff mobilization. Additionally, a normal brain CT on admission doesn’t exclude the risk of elevated ICP nor the development of elevated ICP [12].

In order to overcome these difficulties, non-invasive monitoring techniques have been developed [13] using transcranial Doppler (TCD) [14-17], an automatic quantitative pupillometer [18,19], ocular ultrasound for optic nerve sheath diameter (ONSD) measurement [20,21], and optic nerve disk elevation assessment [22,23]. While these techniques are available, their availability within hospitals, the availability of training staff, the availability of staff to perform these procedures, and the costs to sustain these procedures are unknown. The Brussels Consensus of non-invasive ICP monitoring (B-ICONIC) suggests a mixed-method approach, accumulating data from literature reviews and an in-person consensus, and recently provided recommendations for using non-invasive ICP monitoring and developed algorithms for either escalation or de-escalation of treatments based on clinical findings, with or without imaging, and tool availability. Based on cumulative studies, there is a strong recommendation to combine automatic pupillary assessment with the Neurological Pupil Index (NPi) from the pupillometer, pulsatility index (PI) from Transcranial color duplex (TCD)/ TCC Doppler (TCCD), and ONSD to non-invasively estimate the ICP [4]. However, each of them alone has only moderate positive predictive value for increased ICP, making their use limited in daily clinical practice. 

The aim of the present study is to validate a pragmatic multimodal approach of non-invasive monitoring tools for ICP detection.

## Materials and methods

This was a retrospective study of adult patients (aged ≥ 18 years) who were hospitalized in the neuroscience intensive care unit (NSICU) of The Mount Sinai Hospital, New York, United States, over a period of four years between March 1, 2019, and June 30, 2022. The study was approved by the Institutional Review Board of the Mount Sinai School of Medicine in accordance with Mount Sinai’s Federal Wide Assurances (FWA#00005656, FWA#00005651) to the Department of Health and Human Services (Project: STUDY-21-01580).

Study population

Included patients required EVD placement for both ICP measurement and management, independent of the initial pathology. Exclusion criteria applied to patients younger than 18 years old, or if the patient did not have an EVD, or if TCD or ocular ultrasound were not simultaneously performed. Clinical characteristics and demographics were collected through our electronic medical record. Prior to EVD placement, risks including coagulopathies due to acute liver failure or other etiologies were evaluated by involved neurosurgeons and neurointensivists, and EVDs were not placed in patients deemed high risk of hemorrhage from EVD placement. Bilateral ONSD and assessed ONDE utilizing ocular ultrasound, bilateral MCA PI utilizing transcranial Doppler, and pupillary reactivity with or without a pupillometer were assessed as part of our multimodal assessment. We assessed the correlation and agreement of these values with the ICP measured by the EVD.

EVD, ICP management, and primary outcome

All included patients had daily assessments of the EVD level appropriateness and reliability, based on hourly ICP measurements and waveform compliance, and hourly neurological clinical examination off sedation by a certified neurointensivist and intracranial imaging (when considered necessary). For the purpose of this study, the EVD level was dichotomized to 0-5 and 6-10 cm above the foramen of Monro.

According to our institutional protocol, these patients are eligible to get a daily ocular ultrasound for ONSD measurement and ONDE assessment, which we obtained simultaneously, pupil assessment (clinical reaction to light with or without automatic pupillometer), and TCD for bilateral MCA PI calculation. This multimodal assessment is usually repeated in cases of sustained ICP ≥ 15 mmHg. Patients with no acoustic windows or ocular trauma do not get TCD or ocular ultrasound, respectively, and are not included in our cohort since multimodal evaluation was not feasible.

Patients with ICP ≥ 20 mmHg for more than 10 minutes would undergo targeted ICP treatment as per our standardized protocol. Patients with sustained ICP ≥ 15 mmHg are considered high risk and monitored more closely [24-26].

For the purpose of this study, patients were dichotomized into two groups based on ICP measurement: ICP < 15 mmHg and ICP ≥ 15 mmHg, since an ICP ≥ 15 mmHg is abnormal, and patients with acute brain injury and persistent ICP ≥ 15 mmHg are considered high risk and require more frequent neurological assessments, even if no immediate treatment is commenced.

Data collection and variable definitions

Ocular ultrasound was obtained with the 13-6 MHz linear probe (FUJIFILM Sonosite, Inc., Bothell, Washington, United States) placed on the upper eyelid in a horizontal direction, with the patient in a supine position and head elevated at 30 degrees. The optic nerve was seen longitudinally to the orbit, and its diameter was measured at 3 mm behind the retina [27]. Bilateral ONSD was obtained and averaged; a mean diameter > 5 mm was considered abnormal.

**Figure 1:**
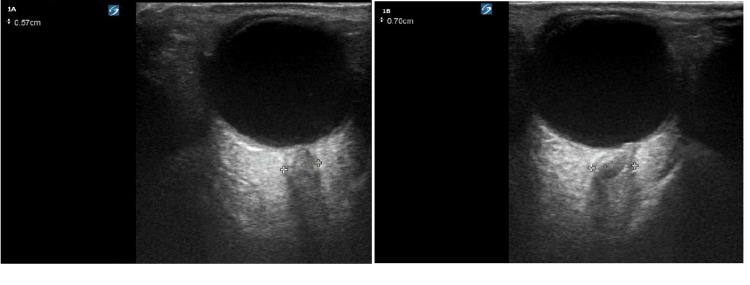
(A) Abnormal ONSD demonstrating diameter 0.57. (horizontal line); (B) Abnormal ONSD demonstrating diameter of 0.7 cm (horizontal line) ONSD: optic nerve sheath diameter

ONDE into the vitreous cavity was also assessed during ocular ultrasound. Disk height was obtained by measuring the distance between the peak of the disk and its intersection with the posterior surface of the globe. A unilateral or bilateral measurement > 0.6 mm was considered abnormal [22-24]. An example of ONSD is shown in Figure 1.

Bilateral MCA PI was assessed with the patient in supine position and head elevated at 30 degrees, with the 2 MHz pencil probe (portable transcranial Doppler neural analytics) placed on the transtemporal window, targeting the MCA between 50-60 mm of depth with the appropriate probe angle. PI is automatically calculated and is equal to $\frac{\text{peak~ systolic~velocity (PSV)} - \text{EDV}}{\text{MFV}}$ [28,29]. Bilateral MCA PI was obtained and averaged.

Pupillary reactivity was assessed with and without a quantitative pupillometer (NPi-200; NeurOptics, Irvine, California, United States). Cerebral perfusion pressure (CPP) was calculated during ICP assessment and is equal to mean arterial pressure (MAP) - ICP. Relevant clinical characteristics, including age, gender, weight, temperature, sedative drugs, and EVD level, were collected.

Statistical analysis

The cohort was analyzed using Fisher’s exact test or Mann-Whitney U-test as appropriate. Specificity, sensitivity, positive predictive value (PPV) and negative predictive value (NPV) were assessed for ICP ≥ 15 mmHg, using an exact binomial 95% confidence interval (CI), for mean ONSD > 5 mm, unilateral or bilateral presence of ONDE and mean MCA PI > 1.2 To evaluate the performance of all above-mentioned variables, unweighted accuracies and areas under receiver operating characteristic (ROC) curves were calculated. Calculations were performed with GraphPad Prism 8 software (Dotmatics, Boston, Massachusetts, United States). Significance was set at p < 0.05.

## Results

A total of 56 measurements were collected from 40 patients. Table 1 demonstrates the patient’s clinical characteristics. Clinically significant characteristics for ICP < 15 mmHg vs ICP >15 mmHg included age (59.6 vs 40.7, respectively, p = 0.0001), bilateral unreactive pupils (0/44 (0%) vs 2/12 (16.67%) readings, respectively, p = 0.0429), unilateral or bilateral ONDE (2/44 (4%) vs 4/12 (33.3%) readings, respectively, p = 0.0155), and CPP (95.9 mmHg vs 67.1 mmHg, respectively, p = 0.0022).

**Table 1:** Clinical characteristics of the studied cohort. Note that because of multiple measurements in some patients, the same patient may be represented more than once. ONSD: optic nerve sheath diameter; MCA: middle cerebral artery; PI: pulsatility index; ICP: intracranial pressure; EVD: external ventricular drain; CPP: cerebral perfusion pressure

Characteristics	ICP <15 (n= 44)	ICP ≥15 (n= 12)	P value	Test
Age (years), mean	59.6 (n= 30)	40.7 (n=11)	0.0001	U-test
Female sex, n	18 (n=30)	7 (n=11)	>0.999	Fisher
Weight (kg), mean	80.85	86.07	0.34	U-test
ONSD (cm), mean	0.506	0.537	0.27	U-test
ONSD Right (cm), mean	0.5068	0.5247	0.567	U-test
ONSD Left (cm), mean	0.5061	0.5498	0.1808	U-test
MCA PI R/L, mean	1.099	1.178	0.5009	U-test
MCA R, mean	1.098	1.173	0.5285	U-test
MCA L (mean)	1.076	1.183	0.4218	U-test
Bilateral unreactive pupils	0/44 (0%)	2 (16.67%)	0.0429	Fisher
Any Optic nerve disk elevation (unilateral or bilateral), n (%)	2 (4.55%)	4 (33.33%)	0.0155	Fisher
EVD level 0-5, n (%)	16 (36.36%)	6/6 (100%)	0.5084	Fisher
Sedation (yes/no), n (%)	9 (20.45%)	5 (41.67%)	0.1506	Fisher
Temperature (°C), mean	37.38 (Missing 3)	36.23 (Missing 1)	0.0054	U-test
CPP (mmHg), mean	95.14 (Missing 2)	67.70 (Missing 2)	0.0022	U-test

Table 2 illustrates the correlation and predictive characteristics for ICP ≥ 15 mmHg based on singular variables. ONSD > 5 mm has excellent sensitivity (0.91, 95%CI 0.61 - 0.99) and NPV (0.95, 95%CI 0.72-0.99) while ONDE has excellent specificity (0.95, 95%CI 0.84-0.99) for association with ICP≥15 mmHg. Similarly, bilateral unreactive pupils show excellent specificity (1, 95%CI 0.92-1) and PPV (1, 95%CI 0.16-1) for rising ICP.

**Table 2:** Prognostic value for ICP ≥15 mmHg ONSD: optic nerve sheath diameter; MCA: middle cerebral artery; PI: pulsatility index; PPV: positive predictive value; NPV: negative predictive value; ICP: intracranial pressure

Characteristic	Sensitivity	Specificity	PPV	NPV
ONSD > 5 mm (unilateral or bilateral)	0.91 (95%CI 0.61 – 0.99	0.41 (95%CI 0.26-0.56)	0.29 (95%CI 0.24-0.36)	0.95 (95% CI 0.72-0.99)
Optic nerve disk elevation (unilateral or bilateral)	0.33 (95%CU 0.09-0.65)	0.95 (95%CI 0.84-0.99)	0.67 (95%CI 0.292 – 0.91)	0.84 (95%CI 0.78-0.89)
MCA PI > 1.1(Unilateral or bilateral)	0.58 (95%CI 0.28-0.85)	0.41 (95%CI 0.26-0.57)	0.21 (95%CI 0.14-0.32)	0.78 (95%CI 0.63-0.89)
MCA PI > 1.2 (Unilateral or bilateral)	0.58 (95%CI 0.28-0.85)	0.59 (95%CI 0.43-0.74)	0.28 (95%CI 0.18-0.41)	0.84 (95%CI 0.72-0.91)
Bilateral unreactive pupils	0.17 (95%CI 0.02-0.48)	1 (95%CI 0.92-1)	1 (95%CI 0.16-1)	0.81 (95%CI 0.77-0.85)

Testing a multimodal approach for ICP ≥ 15 mmHg prediction, the presence of two or more variables among ONSD > 5 mm, presence of ONDE, MCA PI > 1.2 and bilateral unreactive pupils had only a moderate specificity and NPV for ICP ≥ 15 mmHg and this was confirmed by the area under the curve, ROC (0.64 (95%CI 0.50-0.77)) (Table 3).

**Table 3:** Prognostic value of the multimodal approach for ICP ≥15 mmHg* *presence of 2 or more of the following variables: ONSD >5 mm, presence of ONDE, MCA PI >1.2, and bilateral unreactive pupils ONSD: optic nerve sheath diameter; PPV: positive predictive value; NPV: negative predictive value; ICP: intracranial pressure; ROC: receiver operating characteristic

Parameters	ICP ≥15
Sensitivity	0.67 (95%CI 0.35-0.90)
Specificity	0.64 (95%CI 0.48-0.78)
PPV	0.33 (95%CI 0.22-0.47)
NPV	0.88 (95%CI 0.75-0.94)
Area under the ROC	0.64 (95%CI 0.50-0.77)

In testing the multimodal approach with the presence of 3 or more variables (Table 4) among ONSD > 5 mm, presence of ONDE, MCA PI > 1.2, and bilateral unreactive pupils demonstrated excellent specificity (1, 95%CI 0.92 - 1) and PPV (1, 95%CI 0.39-1) for prediction of ICP ≥ 15 mmHg.

**Table 4:** Prognostic value of the multimodal approach for ICP ≥15 mmHg* *presence of 3 or more of the following variables: ONSD >5 mm, presence of ONDE, MCA PI >1.2, and bilateral unreactive pupils ONSD: optic nerve sheath diameter; PPV: positive predictive value; NPV: negative predictive value; ICP: intracranial pressure; ROC: receiver operating characteristic

Parameters	ICP ≥15
Sensitivity	0.33 (95%CI 0.09-0.65)
Specificity	1.00 (95%CI 0.92-1.00)
PPV	1.00 (95%CI 0.39-1.00)
NPV	0.85 (95%CI 0.79-0.89)
Area under the ROC	0.85 (95%CI 0.74-0.94)

## Discussion

These findings suggest that a multimodal approach, especially with several types of simple noninvasive tests, can estimate an ICP value ≥ 15 mmHg. To the best of our knowledge, this is the first time a multimodal approach has been clinically assessed to be utilized for this ICP goal. Previous studies have shown that multimodal combinations with ONSD and TCD may be utilized to estimate elevated ICP; however, the value was not defined as compared to non-elevated ICP [11], and historically, the majority of these studies have included TBI, whereas our cohort had various intracranial pathologies.

General guidelines dictate to initiate treatment when ICP is persistently ≥ 20 mmHg, whereas Brain Trauma Foundation guidelines recommend maintaining an ICP < 22 mmHg [2]. However, an ICP ≥ 15 mmHg is abnormal [25,26], and patients with acute brain injury and persistent ICP ≥ 15 mmHg are considered high risk and require more frequent neurological assessments, even if no immediate treatment is commenced. Accurate assessment of increased ICP is crucial for patient management stratification. Even though an invasive ICP monitor is the gold standard [2,3], the validation of a noninvasive ICP monitor model is necessary, especially for patients who cannot tolerate invasive monitoring. Many isolated noninvasive ICP monitor tests have been used since the 1970s [30]; however, each one alone has only shown moderate accuracy.

The recently published B-ICONIC study reviewed and focused on accuracy for ICP estimation with various non-invasive modalities and their respective negative and positive predictive values, either individually or in the multi-modal approach [4]. Our novel study further defined specific ICP thresholds in addition to identifying lower thresholds for recognizing elevated ICP. Whereas our study used a threshold of ICP ≥ 15 mmHg, a specific ICP cut-off value was not pre-determined in B-ICONIC [4]. Concerning ONSD, the best cut-off of 5.9 mm was defined in B-ICONIC, and ONSD combined with PI and non-invasive ICP (nICP)-TCD were considered as acceptable markers to raise suspicion of excluding increased ICP. Additionally, when utilizing automated pupillometry, NPI < 3 were recommended for use with at least one other tool to assess the suspected presence of elevated ICP, and NPI > 4 to rule out the presence of elevated ICP. Our findings utilized a lower mean ONSD cutoff of > 5 mm, which was found to be an excellent predictor of ICP ≥ 15 mmHg and high NPV for ICP ≥ 15 mmHg when utilized with other modalities. Additionally, while we did not utilize automated pupillometry, bilaterally unreactive pupils had excellent specificity and PPV for elevated ICP, and when combined with ONDE and PI > 1.2, demonstrated excellent sensitivity and specificity for estimating ICP ≥ 15 mmHg. For TCDs, B-ICONIC suggested considering the threshold of PI of 1.3 in conjunction with a diastolic flow velocity (FVd) <20 cm/second as a threshold for considering low CBF potentially associated with high ICP or for excluding it [4]. Our study utilized a lower threshold PI of 1.2, and when utilized in conjunction with ONDE and bilateral unreactive pupils had excellent sensitivity and specificity for correlation with ICP ≥15 mmHg. Additionally, we included ONDE in our multimodal approach, which was not included in previous studies.

Our findings show an excellent specificity of the presence of ONDE for ICP ≥15 mmHg. It is well known that elevated ICP causes various degrees of optic disk swelling, classically seen on fundoscopy. However, fundoscopy may require pharmacological mydriasis (which will preclude frequent pupillary assessment) and, when performed by nonspecialists, may have inaccurate results [31]. Recent studies have used ocular ultrasound for ONDE assessment; Teismann et al. compared sonographic findings with fundoscopy and found a good sensitivity for optic disc height greater than 0.6 mm, with excellent specificity but diminished sensitivity for height greater than 1 mm [23]. For the present study, optic disc height > 0.6 mm, unilateral or bilateral, was considered abnormal and indicative of increased ICP. We acknowledge that ONDE can occur in a variety of diseases, such as multiple sclerosis, infiltrative optic nerve disease (lymphoma, sarcoidosis, infectious process), idiopathic intracranial hypertension, and malignant systemic hypertension. However, none of the patients included in this study suffered from any of the above-mentioned conditions. 

Ocular ultrasonography is also already widely used for ONSD estimation in the setting of suspected high ICP. The optic nerve is surrounded by the subarachnoid space and, hence, the optic nerve sheath is distensible and reflects CSF pressure variations, which influence the ONSD with fluctuations in the anterior retrobulbar compartment, about 3 mm behind the globe [11,32,33]. A simple bedside tool, ocular ultrasonography is inexpensive, has high reproducibility of measures with careful training, and does not require patient transport [12]. ONSD greater than 4.5-5.5 mm generally correlates with ICP greater than 20 mmHg [20,21,34], even though one study has shown very poor accuracy [35]. Our findings show relatively good sensitivity and specificity of mean ONSD > 5 mm as an estimate of ICP ≥15 mmHg, and a high NPV for ICP ≥ 15 mmHg when utilized as a multimodal approach. The number of patients included in our study is relatively low; therefore, further validation of different cut-off values with ICP measurements was not feasible. Similarly to ONDE, increased ONSD may be associated with several other conditions; however, none of our patients suffered from any concomitant disease affecting the optic nerve.

The use of TCD-measured flow velocities for ICP estimation was first introduced in the 1980s by Klingelhofer et al. [16,17]. Increased ICP affects blood flow velocity in the cerebral vasculature, particularly in major cerebral vessels, and changes in cerebral blood flow include decreased FVd and increased PI [36,37]. Various TCD-derived parameters have been used to assess ICP; however, the MCA PI is the most commonly used [30,38]. A cut-off value of PI ≥ 1.26 is generally a reliable predictor of ICP ≥ 20 cmH_2_0 [14]; however, many criticisms about this correlation exist [38-40]. Of note, the recent IMPRESSIT-2 (Invasive versus noninvasive Measurement of intracranial PRESSure in brain Injury Trial 2) trial demonstrated that TCD had a high NPV in ruling out elevated ICP, particularly when invasive modalities are not available; the optimal ICP was >22 mmHg for elucidating this conclusion [41]. In our study, an MCA PI cut-off > 1.2 had good sensitivity and moderate specificity for predicting ICP ≥15 mmHg, which is equivalent to 20.3 cmH_2_0. Performing focused TCD by the bedside provides quick results, with a minimum level of experience being necessary to provide good insonation through the acoustic window. Additionally, the PI is favored because it is unaffected by factors such as angle of insonation [13]. However, a high PI may be falsely high in patients with increased vascular resistance due to intracranial atherosclerosis [42], making relative changes in PI an interesting future parameter to study for predicting increased ICP. Additionally, the B-ICONIC cutoff of 1.28 was not utilized in our study due to the relatively low number of patients in our study.

Changes assessed either automatically or visually in pupillary reactivity [19], constriction velocity, and shape to an oval pupil, have been used as indirect markers of increased ICP without clear ICP cut-off values. However, a study by Chen et al. did demonstrate the NPi as an early indicator of increased ICP [10]. In our study, almost all patients had reactive pupils to light (either with quantitative or visual evaluation), without anyone experiencing a change in pupillary shape; thus, these parameters were not included in our model. This supports the idea that pupillary reactivity is a late change in elevated ICP and should not be relied upon as a sole measure of ICP. For our study, NPi assessment was not utilized, and rather, we utilized reactivity presence or absence, since not everyone was being assessed with an automatic pupillometer.

Electroencephalogram (EEG) has also been used for ICP estimation; however, specific software for automated analysis is required, and as of now, there is no validated correlation model of EEG features and ICP values. Other modalities have been used, such as anterior fontanelle pressure monitoring, skull elasticity, venous ophtalmodynamomatery, tympanic membrane displacement, tissue resonance analysis, near-infrared spectroscopy, tonometry, acoustoelasticity, acoustic signal generation devices, otoacoustic emissions, and many others [10,30]. However, their application in daily clinical practice is still limited, and further study is required.

This study has some limitations. Even though this model appears accurate for estimating ICP greater than 15 mmHg, the number of included patients is relatively low. This is a single-center pragmatic study, and further validation is necessary in outside facilities. We did not look for multiple cut-offs for different ICPs, but rather dichotomized ICP into “concerning” versus “not concerning.” Dichotomizing further into groups including > 20 mmHg, > 15 mmHg (concerning) versus non-concerning would be a good future comparison into the predictability of this model at different ICP. Finally, the physicians who performed all the examinations were not blinded to any of the ICP measurements; however, given that treatment was not affected based on these measurements, we do not believe that not being blinded influenced the data interpretation. Finally, we relied on the EVD to accurately demonstrate elevated ICP. If the pressures for any patients were falsely low due to inaccuracy of the EVD, this would affect the interpretation as well; however, all included patients had reliable ICP waveforms on bedside monitoring.

## Conclusions

In the presented model, we included data from simple, accessible tests in the Neurosciences Intensive Care Unit, which require minimal training to learn, to accurately estimate higher ICP. The presence of 3 or more variables among: ONSD > 5 mm, presence of ONDE, MCA PI > 1.2, and bilateral unreactive pupils is associated with an ICP ≥ 15 mmHg. Taken together, our findings pragmatically validated a multimodal bedside approach utilizing these simple, widely available tools, and larger patient cohorts exploring these multimodal models would be beneficial to understand which modalities, when used together, would most accurately predict elevated ICP and eventual integration into clinical protocols that could be widely utilized within hospital systems. Similarly, efficacy from a cost and training perspective could be effectively evaluated once these modalities are more widely available and utilized.
